# Evaluation the role of quinoa seeds in attenuation the brain cellular senescence and aging induced by D-galactose and γ-radiation in rats: insights into autophagy, telomerase activity, amyloid-β and tau proteins

**DOI:** 10.1007/s10787-026-02377-1

**Published:** 2026-09-19

**Authors:** Merehan Alaa-ElDin Mohamed, Mustafa M. M. Elbakry, Nermeen M. Elbakary, Said S. Moselhy, Youssef A. Said

**Affiliations:** 1 Biochemistry Department, Faculty of Science, Ain Shams University, Cairo, Egypt; 2 Faculty of Biotechnology, October University for Modern Sciences and Arts (MSA), Cairo, Egypt; 3 Radiation Biology Department, National Centre for Radiation Research & Technology, Egyptian Atomic-Energy Authority, Cairo, Egypt

**Keywords:** Brain cellular senescence, Aging, Autophagy, Oxidative stress, Quinoa seeds, Telomerase, Rats

## Abstract

The gradual loss of cognitive function, manifested by impaired learning and spatial memory, is a hallmark of brain senescence during aging. However, effective natural interventions targeting multiple mechanisms involved in aging-related neurodegeneration remain limited. This study assessed the possible protective efficacy of quinoa seed powder (QSP) against D-galactose (D-gal)- and γ-radiation-induced brain senescence in rats. Fifty male albino rats (n = 10/group) were randomly allocated into five experimental groups. Aging was induced by D-gal administration and fractionated whole-body γ-radiation (1.5 Gy/week for four consecutive weeks; total dose 6 Gy). Behavioral performance, oxidative stress biomarkers, telomerase activity, autophagy markers, neurodegenerative proteins, histopathological alterations, antioxidant activity of quinoa ethanolic extract (QEE), and molecular docking were evaluated. QSP significantly (*P* < 0.05) attenuated oxidative stress, as evidenced by decreased malondialdehyde levels and increased catalase activity and reduced glutathione content. Furthermore, QSP significantly reduced amyloid-β 1–42 (Aβ42) and Tau protein accumulation, increased telomerase activity, and enhanced autophagy-related modulation through upregulation of microtubule-associated protein 1 light chain 3 beta (LC3B) and downregulation of mammalian target of rapamycin (mTOR) expression. In vitro analysis showed that QEE exhibited potent antioxidant activity, with EC50 values of 16.67 and 23.01 mg/mL for TAC and FRAP, respectively, and an IC_50_ value of 5.91 mg/mL against DPPH radicals. Docking analysis showed that kaempferol exhibited the most favorable binding affinities among the tested quinoa constituents toward mTOR (− 7.579 kcal/mol) and catalase (− 7.286 kcal/mol), with short-timescale molecular dynamics analyses providing additional structural support for the predicted interaction profiles. These findings suggest that QSP exerts multi-target neuroprotective effects through attenuation of oxidative stress and modulation of autophagy-related pathways, supporting its potential as a natural strategy for mitigating aging-associated neurodegenerative changes.

**Graphical abstract:**

**Figure:**
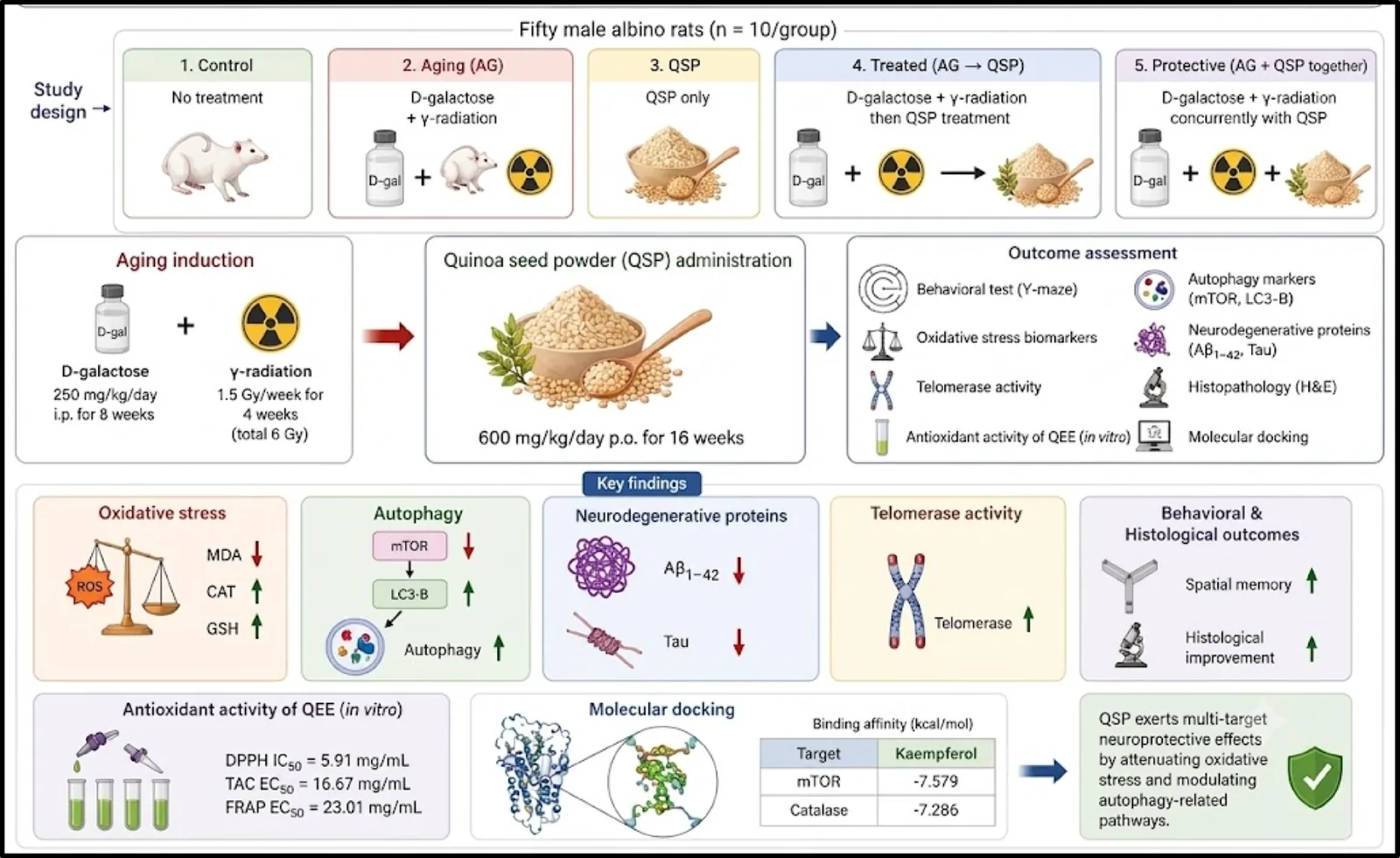

**Supplementary Information:**

The online version contains supplementary material available at https://doi.org/10.1007/s10787-026-02377-1.

## Introduction

Aging is process that involves a gradual decline in organ function and an increasing vulnerability.

to disease. The brain's high energy demand and post-mitotic nature make it particularly vulnerable to age-related structural and cognitive changes (Jin and Cai 2023). The most popular method for induction of the aging process in the animals by using the D-gal that accelerated brain-aging (Martinovic et al. 2023). D-gal administration in animals can produce several signs of brain aging, including cognitive impairment, increased oxidative stress, mitochondrial dysfunction, and apoptosis. In addition, D-gal-induced mitochondrial DNA damage and dysfunction, impaired respiratory function and reduced ATP production (Ma et al. 2024; Martinovic et al. 2023). Moreover, ionizing radiation accelerated aging significantly, especially γ -radiation caused DNA breaks, genomic instability, excessive formation of reactive oxygen species (ROS) and mitochondrial dysfunction^4^. These changes increase the incidence of inflammation and age-related cellular damage by activating cellular senescence pathways and promoting the senescence-associated secretory phenotype. As a result, ɤ-irradiated animals are frequently employed as an experimental model of accelerated aging (Ibragimova et al. 2024).

Protection by natural products against these cellular changes during aging due to their active ingredients are the main targets of many research to provide ana alternative or complementary safe supplement. Quinoa (*Chenopodium quinoa*) is an annual herbaceous flowering plant belonging to the genus *Chenopodium*. It is used a dietary supplement to enhance human health, since it contains minerals, polyphenols, proteins, dietary fibers, amino acids, unsaturated fatty acids, and other active ingredients (Xi et al. 2024). Previous study demonstrated that quinoa seeds possess anti-oxidative, anti-inflammatory, immunomodulatory, anticarcinogenic, prebiotic and anti-obesity properties (Mu et al. 2023). Because of its high nutritional content, quinoa has gained popularity as a functional food in recent years. Apart from its beneficial nutritional qualities, its bioactivity enables expanded used as complementary supplement in medicine (El Hazzam et al. 2020). Quinoa’s health benefits and grain color diversity greatly depends on its polyphenols content, a class of secondary metabolites with several physiological characteristics such as; antibacterial, anti-inflammatory, anticancer, normoglycemic and antioxidant qualities (Zhang et al. 2024). Nowadays, it has been demonstrated that quinoa contains over 20 different phenolic compounds, the majority of which are phenolic acids such as; ferulic acid, vanillic acid, caffeic acid and their derivatives, while flavonoids are mostly found in the glycoside form of kaempferol and quercetin (Ng and Wang 2021). Notably, linoleic acid represents the primary polyunsaturated fatty acid in quinoa, accounting for 49.0–56.4% (Vilcacundo and Hernández-Ledesma 2017). Linoleic acid is an essential omega-6 polyunsaturated fatty acid involved in lipid metabolism and brain function. Alterations in circulating linoleic acid levels have been associated with changes in mitochondrial bioenergetics and cognitive status in individuals with Alzheimer’s dementia (Amick et al. 2022). Quinoa has demonstrated significant therapeutic potential, with studies linking its consumption to improved cardiovascular health and reduced risk factors (Li et al. 2024). Additionally, it exhibits neuroprotective and anti-stress effects, successfully preventing memory deficits and stress-induced hippocampal damage in animal models (Souza et al. 2020). Although quinoa seeds are recognized for their general nutritional and antioxidant potential, their mechanistic role in mitigating multifactorial brain senescence remains poorly understood. To date, no study has investigated the therapeutic effect of QSP on a dual-induced model of neurodegeneration triggered by chronic metabolic stress (D-gal) and ionizing radiation. The novelty of the current study lies in providing the first comprehensive in vitro, in vivo, and in silico framework based demonstrating how QSP exerts multi-targeted neuroprotective effects specifically via oxidative stress, telomerase activity, clearing pathogenic Tau and Aβ proteins, and modulation of autophagy-related markers, including mTOR and of LC3B.

## Materials and methods

### Preparation of quinoa seed powder (QSP) and quinoa ethanolic extract (QEE)

Quinoa seeds (white quinoa seeds, 100% organic, code F070289) were purchased from Imtenan Company, Cairo, Egypt, washed repeatedly with cold water to remove saponins and then air-dried at room temperature for 24 h. The dried seeds were then finely ground using a laboratory mill to prepare QSP as previously described by Abugoch (2008). QEE was prepared from QSP according to the reported method of Park (2017) through maceration in 70% ethanol at room temperature for 24 h. with continuous shaking on orbital shaker, followed by centrifugation at 3500 *x*g for 10 min. at 5 °C), then filtered through Whatman No. 1 filter paper. The activity of supernatant was evaluated in vitro study as DPPH radical scavenging assay, total antioxidant capacity and ferric-reducing antioxidant power assay.

### In vitro studying the activity of QEE

- DPPH radical scavenging activity

Free radical scavenging activity of QEE was evaluated using the 2,2,-diphenyl-picrylhydrazyl (DPPH) according to the method of Blois (1958). A volume of 3.0 mL of a freshly prepared QEE at different concentrations (5–30 mg/mL) was mixed with 1.0 mL of ethanolic DPPH solution (0.1 mM), followed by incubation at 25 °C for 30 min. in dark. Absorbances were measured against absolute ethanol as blank (λ_max_ 519 nm). Ascorbic acid was employed as the standard positive control. IC_50_, which is the concentration required to achieve 50% radical scavenging activity, was calculated from the graph of DPPH free radical scavenging activity percentage versus different QEE concentrations (5–30 mg/mL). The scavenging activity was calculated using the formula:

$$DPPH\, scavenging\, activity \left(\% inhibition\right)=\left(\frac{Ac-At}{Ac}\right)\times 100$$

where A_c_ is the absorbance of the control (pure DPPH). A_t_ is the absorbance of the test solution.

b. Total antioxidant capacity (TAC)

The total antioxidant capacity of QEE was determined using the phosphomolybdenum method, as described by Ahmed et al. (2012). A volume of 300 *µ*L of QEE ranging in concentration (5–30 mg/mL) was mixed with 3.0 mL of the reagent solution (0.6 mM sulfuric acid, 28 mM sodium phosphate and 4.0 mM ammonium molybdate). The tubes were covered and incubated for 90 min. in a hot water bath (95 °C). After the tubes were cooled, absorbances were measured against the sample' solvent as a blank (λ_max_ 695 nm). The antioxidant activity was reported in mg/mL of ascorbic acid equivalents (AAE). Ascorbic acid was prepared at different concentrations (10–500 µg/mL) as a reference standard. A linear calibration curve (*y* = 0.0018*x* + 0.0094; R^2^ = 0.9976) was applied to assess the extracts' antioxidant power.

c. Ferric-reducing antioxidant power (FRAP)

The ferric-reducing power of QEE was investigated according to the method of (Oyaizu 1986). Briefly, 1.0 mL of different QEE concentrations (5–30 mg/mL) was mixed with 2.5 mL of potassium ferricyanide (1.0%) and incubated at 50 °C for 30 min. Then, 2.5 mL of trichloroacetic acid (10%) was added to the mixture and centrifuged (10 min. at 3000 xg). A volume of 2.5 mL of the supernatant was mixed with 2.5 mL distilled water and 0.5 mL freshly prepared ferric chloride solution (0.1%). The absorbances were measured at 700 nm. Increased absorbance of the reaction mixture indicated increased reducing power. The same circumstances were applied to ascorbic acid (5–100 µg/mL) as a positive control. Reducing power was reported in mg/mL of ascorbic acid equivalents (AAE). The linear calibration curve (*y* = 0.0073*x−*0.0063; R^2^ = 0.9989) was applied to assess the extract's reducing power.

### Experimental animal design

Fifty male Wistar albino rats (120 ± 10 g) were purchased from the animal farm of the Egyptian Holding Company for Biological Products and Vaccines (VACSERA), Helwan, Cairo, Egypt. Animals were housed in a polycarbonate cage at (21–22 °C), Fed on a standard pellet diet and had free access to tap water. The experiment was performed at the animal house of the National Centre for Radiation Research & Technology, Egyptian Atomic Energy Authority, in accordance with institutional guidelines for the care and use of laboratory animals. The research protocol was reviewed and approved by the Research Ethics Committee, Faculty of Science, Ain Shams University (Approval No. ASU-SCI/BIOC/2025/6/2; reviewed on 15 June 2025). The study was conducted in accordance with the ARRIVE 2.0 guidelines and relevant regulations governing animal experimentation.

**Fig. 1:**
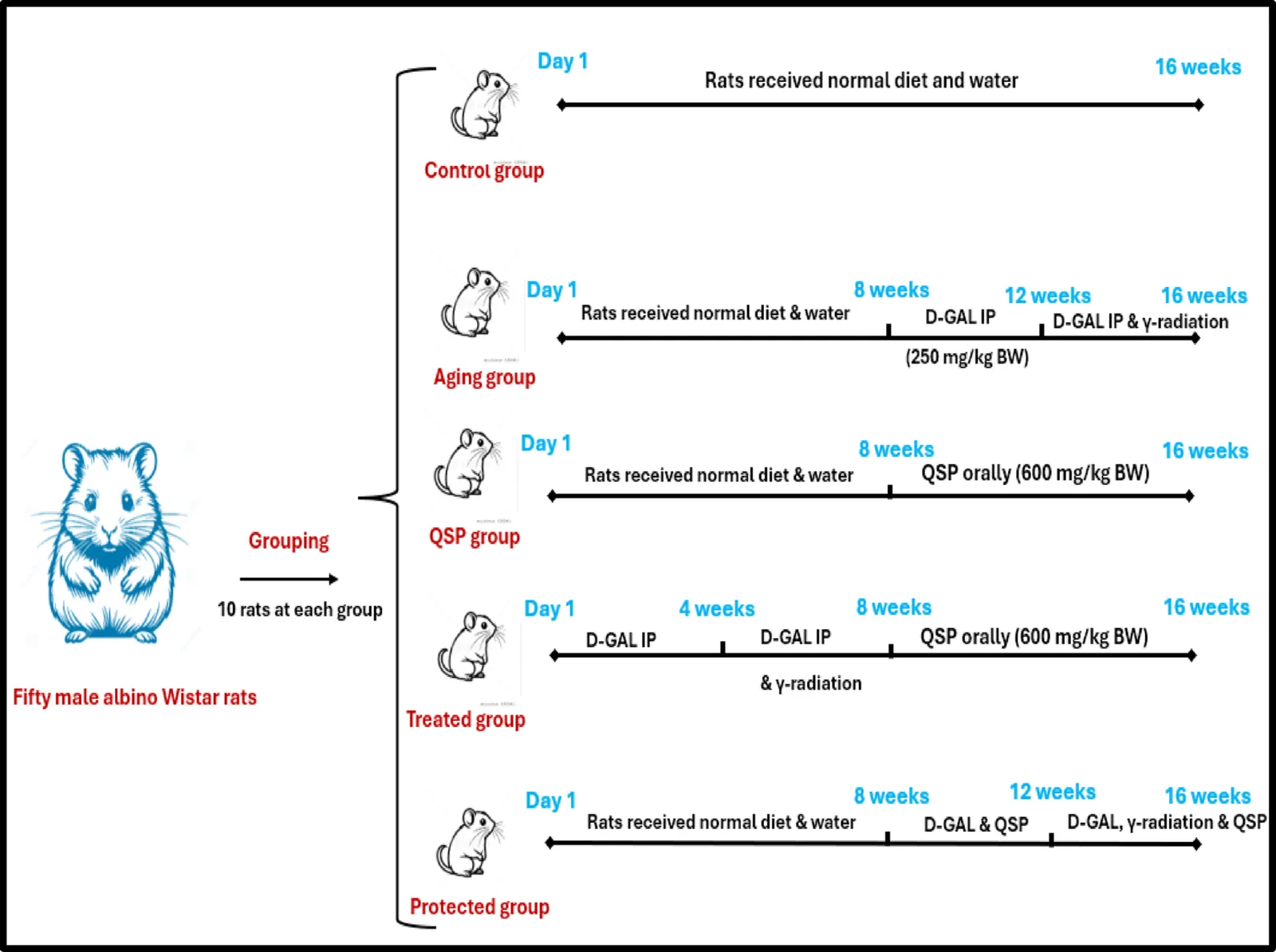
Schematic representation of experimental design. Fifty male albino rats were randomly allocated into five experimental groups: control, QSP, aged, treated, and protective groups (n = 10/group)

Figure 1 depicts the timeline of the applied experimental protocol. After 10 days of adaptation, rats were randomly allocated into 5 groups (n = 10) as follows:

*Group 1 (Control)* Rats were kept untreated as control for 16 weeks.

*Group 2 (Aging)* Rats received D-gal (250 mg/kg bw) in sterile saline intraperitoneally daily for 8 weeks according to the modified method of Ma et al. (2021), and exposed to γ-radiation (^137^Cs, 1.5 Gy/week for a total of 6 Gy/animal) for 4 weeks starting from the fifth week of D-gal induction according to Yakunchikova et al. (2024).

*Group 3 (QSP)* Rats were received quinoa seeds powder (QSP) (600 mg/kg bw) suspended in normal saline intragastric daily for 8 weeks according to Arshad et al. (2024).

*Group 4 (Treated)* Rats received D-gal and were exposed to **γ-**radiation for 8 weeks then treated with QSP for 8 weeks.

*Group 5 (Protected)* Rats D-gal and exposed to **γ-**radiation for 8 weeks as in group-2 with concomitant administration of QSP.

### Behavioral examination using Y-maze

At the end of the experimental period (16 weeks), Y-maze test was performed to assess the short-term spatial memory of animals as a type of cognition. The apparatus was made up of three equal-length opaque arms (35 × 35 × 35 cm^3^) arranged at 120° angles. After being placed in the middle of the maze, each rat was given five minutes to move freely. When the animal’s four limbs entered the same arm, it was recorded as an arm entry. Three successive entrances into different arms were considered a successful alternation (Lan et al. 2025b).

$$Spontaneous\, alteration \left(\% \right)=\left(\frac{number\, of \,successful \, alternations}{total\, arm\, entries - 2}\right)\times 100$$

### Preparation of brain tissue homogenate for biochemical analysis

After Y-maze test, rats were fasted overnight and euthanized via intraperitoneal injection of a single urethane dose (1.2 g/kg bw). Brain tissue was dissected out and divided into three parts; one part was kept in 10.0% neutral buffered formalin for histopathological studies, the second part was lysed in NP-40 lysis buffer to assess telomeric activity and the third part was homogenized (10.0%) in phosphate-buffered saline (pH 7.4) using a glass tissue homogenizer with a Teflon pestle. The entire brain homogenate was subjected to centrifugation at 18,000× g for 15 min at 4 °C using a Cooler Microfuge Laborzentrifugen (Sigma, Germany) to isolate the cytosolic fraction, which was subsequently collected, aliquoted, and kept at − 20 °C until estimation of the biochemical parameters.

### Assessment of oxidative stress biomarker

GSH content (Cat. No. GR2511), CAT activity (Cat. No. CA2517) and MDA level (Cat. No. MD2529) were assayed in brain homogenate using commercially available colorimetric assay kits purchased from Bio diagnostic, Egypt.

### Evaluation of autophagy biomarkers.

mTOR (Cat. No. E-31091Ra) and LC3B (Cat. No. E-31077Ra) were determined in brain homogenate using rat sandwich ELISA kits purchased from Cloud-Clone Corp (USA).

### Determination of abnormal neural protein fibrils

Brain tissue homogenates in each group were assayed for Aβ42 (Cat. No. E-EL-R1402) and Tau level (Cat. No. MBS700575) using rat sandwich ELISA kits purchased from Elabscience (China) and MyBioSource, Inc. (USA), respectively.

### Determination of telomerase activity

Telomerase activity in brain homogenate was detected by real-time telomeric repeat amplification protocol (RT-TRAP) (Herbert et al. 2006). Briefly, 1 mg of brain tissue was lysed with NP-40 lysis buffer. The total protein content was determined using the Pierce BCA Protein Assay Kit purchased from Thermo Fisher Scientific (USA). Despite differences in protein concentrations among samples, an equal amount of total protein (1.0 µg) from each sample was normalized to a final volume of 2 µL, then added to a reaction mix with a total volume of 20 µL per sample containing 10 µL SYBR Green Master Mix (Qiagen, Germany), 100 ng each of the primers TS (5’-AATCCGTCGAGCAGAGTT-3’) and ACX (5'-GCGCGG(CTTACC)3CTAACC-3'), and RNase/DNase-free H₂O water to bring the final volume to 20 µL. A serial dilution of the A549 cell line, acting as a positive control, was used for making the standard curve. The reaction mixture was incubated at 30 °C for 30 min in the dark for extension of the substrate by telomerase. Then amplified by SYBR Green using a real-time polymerase chain reaction (Agilent Stratagene MX3000P, USA) with the following conditions: 95 °C for 10 min, followed by 40 cycles of 95 °C for 10 s and 60 °C for 60 s. The relative telomerase activity (RTA) was calculated from the standard curve using the following equation: RTA = 10^[(C^_t sample_^–Y^_int_^) / Slope]^

where RTA: Relative telomerase activity. ^C^_t sample_: Cycle threshold of the target sample. ^Y^_int_: Y-intercept of the standard curve.

### Histopathological examination

Formalin-fixed paraffin-embedded brain tissue sections were stained with hematoxylin and eosin (H&E) according to the method reported by (Drury 1983) Histopathological examination was performed in the cerebral cortex and CA1 hippocampal region using an Olympus CX41RF microscope (Olympus Corporation, Tokyo, Japan) equipped with an Olympus Soft Imaging SC100 camera at ×400 original magnification. Representative photomicrographs were captured using a 50 µm scale bar. Histopathological scoring was performed for both brain regions examined.

### Docking studies

The main bioactive compounds from quinoa grains were selected for molecular docking based on the metabolomics data from Younis et al. (2025). Three representative ligands were chosen based on their high abundance and occurrence in the Egyptian quinoa profile: Linoleic acid was selected as the predominant fatty acid from the GC–MS data, Kaempferol was chosen as the core representative flavonoid aglycone, and Ferulic acid was selected as the major phenolic acid identified via LC-QTOF-MS/MS. The 3D structures of these three parents’ compounds (Linoleic acid, Kaempferol, and Ferulic acid) were retrieved from the PubChem database to evaluate their binding energies against the target proteins.

In silico molecular docking was performed to investigate the binding affinity and interaction modes of linoleic acid, kaempferol, and ferulic acid against three key target proteins by using AutoDock Vina (version 1.2.5). The crystal structures of the target proteins were retrieved from the RCSB Protein Data Bank (PDB) with the following IDs: mTOR protein (PDB ID: 4jsp) and catalase (PDB ID: 8HID). In addition to the co-crystallized ligands, the ATP-γS-Mg complex (PDB ID: 4jsp) and LLR [(~ {S)}-azanyl-[2-[[3-bromanyl-4-(diethylamino)phenyl]methyl]hydrazinyl]methanethiol] (PDB ID: 8HID) were used as reference ligands, respectively.

*Receptor preparation* The target protein structures were prepared using PyMOL (version 2.5.4), where the co-crystallized ligands and solvent molecules were removed, hydrogen atoms were added, and the prepared protein structures were saved in PDB format (Solo 2021). The prepared receptors were subsequently processed using AutoDockTools (MGLTools) for the addition of Kollman charges and preparation for docking. The docking site and grid box dimensions were defined based on the position of the corresponding co-crystallized ligand (Morris et al. 2009). The grid box dimensions and centers are presented in Table 1.

**Table 1:** The grid box dimensions used for molecular docking

Protein symbol	n.pts (x; y; z)	Center (x; y; z)
mTOR	24; 24; 40	50.001; −1.650; −44.992
Catalase	32; 26; 22	16.070; 8.219; −68.906

*Ligand preparation* The 3D structures of these three parents’ compounds (Linoleic acid, Kaempferol, and Ferulic acid) were retrieved from the PubChem database to evaluate their binding energies against the target proteins. The ligand structures were subjected to energy minimization using Open Babel with the MMFF94 force field for 1000 steps. The minimized ligands were then converted to PDBQT format for molecular docking (O’Boyle et al. 2011).

The prepared ligands were docked into the defined binding sites using AutoDock Vina (version 1.2.5). The molecular interactions between the docked ligands and target proteins were visualized using BIOVIA Discovery Studio Visualizer (version 4.5).

To validate the docking protocol, the corresponding co-crystallized reference ligands were redocked into their respective binding sites. The root-mean-square deviation (RMSD) between the crystallographic and redocked poses was calculated.

### Molecular dynamics analysis

To further assess the short-timescale structural stability of the docked protein–ligand complexes, molecular dynamics simulations were performed for the six protein–ligand complexes using the prepared docked structures. The trajectories were analyzed for protein Cα RMSD, ligand heavy-atom RMSD, per-residue RMSF, radius of gyration (Rg), ligand solvent-accessible surface area (SASA), and protein–ligand hydrogen-bonding interactions. The resulting parameters were calculated as trajectory-averaged values and expressed as mean ± SD.

### Statistical analysis

To control confounding environmental factors and ensure unbiased group assignment, all animals included in the study were of the same strain (Wistar), same sex (males) with matching age (4–6 weeks) and body weight (120 ± 10 g). They were randomly categorized into 5 groups (n = 10) in number-coded cages under the same housing conditions (temperature, aeration and dark–light cycles), and food and water accessibility. Sample size was determined to ensure statistical validity, to tolerate any mortalities that may occur and to soothe intra-individual variations in biology of animals of the same group. Either pre- (aging induction, QSP administration and behavioral evaluation) or post-euthanasia (sample collection, biochemical and histopathological analyses), investigators were completely blinded to the specific group allocations to minimize performance bias. Statistical analysis of data was performed using a one-way analysis of variance (ANOVA). Data were found to be normally distributed and of homogenous variance as per Shapiro–Wilk normality test and Levene’s homogeneity of variances test, respectively. For multiple pairwise comparisons between groups, least significant difference (LSD) post hoc test was performed. All values were reported as mean ± standard deviation and considered statistically significant when *p* ≤ 0.05. All statistical analyses were performed using SPSS software (version 24.0).

## Results

### In vitro antioxidant activity of QEE

#### DPPH radical scavenging activity

DPPH radical-scavenging activity (% inhibition) at different QEE concentrations is presented in Table 2. Data showed that QEE has IC_50_ value of 5.91 mg/mL, compared to the standard ascorbic acid (IC₅₀ = 2.32 mg/mL).

**Table 2:** DPPH radical scavenging activity, total antioxidant activity and ferric-reducing power of QEE

QEE Concentration (mg/mL)	DPPH scavenging activity (% inhibition)	Total antioxidant activity (mg AAE/g)	Ferric-reducing antioxidant power (mg AAE/g)
5	23.30 ± 1.66	6.48 ± 0.10	8.34 ± 0.32
8	65.30 ± 2.78	8.55 ± 0.16	9.93 ± 0.40
10	67.30 ± 0.93	20.16 ± 0.13	12.01 ± 0.30
20	78.30 ± 1.66	23.56 ± 0.49	15.07 ± 0.21
30	87.70 ± 1.37	26.50 ± 1.32	17.53 ± 0.57

QEE: Quinoa ethanolic extract. Data were expressed as mean ± standard deviation (n = 3)

#### Antioxidant activities (TAC and FRAP)

Results in Table 2 indicated that TAC and FRAP of QEE were increasing in a concentration-dependent manner. EC_50_ (half maximal effective concentration) was used to determine antioxidant potency. The EC_50_ of TAC and FRAP of QEE were 16.67 mg/mL and 23.01 mg/mL, respectively, compared to ascorbic acid as a reference standard, whose values were equal to 0.27 mg/mL and 0.069 mg/mL, respectively.

#### Behavior analysis of rats by using the Y-maze

Data obtained in Table 4 showed that spatial working memory was reduced significantly (*p* ≤ 0.001) in the rats injected with D-gal and exposed with ɤ- radiation. However, administration of QSP, either in treated (group-4) or protected (group-5) rats showed a significant improvement (*p* ≤ 0.001) in the spatial memory in comparison with untreated. The treated group is better than protective group.

#### Biochemical analysis in brain homogenates

Data presented in Table 3 indicated a significant decrease (*p* ≤ 0.001) in reduced glutathione (GSH) and catalase (CAT) activity, and a significant elevation (*p* ≤ 0.001) in malondialdehyde (MDA) level in brain tissue homogenate in induced aging rats compared to the control group. The rats given QSP either as therapeutic or prophylactic showed a significant increase in GSH content and CAT activity, accompanied by a remarkable decrease in MDA level in comparison with untreated rats. Compared to the protection group, the treatment group showed a more noticeable rise in GSH level and CAT activity along with a decrease in MDA levels.

**Table 3:** Statistical analysis (ANOVA) for oxidative stress markers in all studied groups

Parameters	Group (1) Control	Group (2) Aging D-gal+ ɤ-radiation	Group (3) QSP	Group (4) Treated	Group (5) Protected
MDA (nmol/g tissue)	1223 ± 7.23^**b**^	2059 ± 6.71^**a**^	1222.50 ± 5.43^**b**^	1406 ± 3.03^**ab**^	1479 ± 4.54^**ab**^
GSH (mg/g tissue)	376.60 ± 15.75^**b**^	49.60 ± 7.31^**a**^	374.30 ± 11.72^**b**^	267.80 ± 4.87^**ab**^	186.50 ± 4.08^**ab**^
CAT (U/g tissue)	124.16 ± 5.30 ^**b**^	36.16 ± 5.38^**a**^	123.60 ± 4.63^**b**^	71.50 ± 4.67^**ab**^	62.83 ± 2.56^**ab**^

MDA: Malondialdehyde. GSH: Reduced glutathione. CAT: Catalase. QSP: Quinoa seeds powder

Data are expressed as mean ± SD (n = 6). *P* ≤ 0.05 was considered statistically significant. ^**a**^ Significantly different from the control group; ^**b**^ significantly different from the aged group

Changes in autophagy-associated markers during aging were presented in Table 4, the induced aging group had significantly higher level of mTOR in brain tissue and lower level of LC3B (*p* ≤ 0.001) than the control group. However, rats’ administration with QSP led to a significant increase (*p* ≤ 0.001) in the LC3B level and a significant decrease (*p* ≤ 0.001) in the mTOR level in both QSP-treated and QSP-protected groups.

**Table 4:** Statistical analysis (ANOVA) for Y-maze, mTOR, LC3B, tau, Amyloid-β and telomerase activity

Parameters	Group (1) Control	Group (2) Aging D-gal+ ɤ-radiation	Group (3) QSP	Group (4) Treated	Group (5) Protected
Y-maze (%)	94.80 ± 5.67^b^	68.7 ± 5.07^a^	92.3 ± 6.08^b^	89.30 ± 6.21^b^	81.80 ± 3.97^ab^
mTOR (pg/mL)	112.00 ± 5.83^b^	307.80 ± 2.71^a^	111.5 ± 4.46^b^	185.66 ± 9.02^ab^	210.60 ± 7.17^ab^
LC3-B (pg/mL)	570.16 ± 12.82^b^	162.33 ± 6.80^a^	565.66 ± 10.42^b^	391.33 ± 8.14^ab^	330.00 ± 7.67^ab^
Tau (pg/mL)	12.20 ± 0.85^b^	51.00 ± 1.41^a^	11.80 ± 0.93^b^	23.70 ± 1.54^ab^	17 ± 0.77^ab^
Amyloid-β (pg/mL)	10.10 ± 0.89^b^	58.50 ± 1.87^a^	9.73 ± 0.77^b^	29.20 ± 1.03^ab^	19.90 ± 0.81^ab^
Telomerase (RTA)	2.30 ± 0.10^b^	0.07 ± 0.01^a^	2.19 ± 0.08^b^	1.10 ± 0.10^ab^	0.84 ± 0.05^ab^

mTOR: Mammalian target of rapamycin. LC3-B: microtubule-associated protein-1 light chain-3 beta. RTA: Relative telomerase activity. QSP: Quinoa seeds powder

Data are expressed as mean ± SD (n = 6). *P* ≤ 0.05 was considered statistically significant. ^**a**^ Significantly different from the control group; ^**b**^ significantly different from the aged group

Changes in neurodegeneration-associated proteins were shown in Table 4, indicated a significant elevation (*p* ≤ 0.001) in the levels of Tau and Aβ42 proteins and a significant reduction (*p* ≤ 0.001) in telomerase activity induced aging by (**γ**-irradiated D-gal) administered rats compared to normal ones. Administration of QSP apparently reversed these abnormalities. The treatment group showed better results than protection group.

#### Histopathological studies

**Fig. 2:**
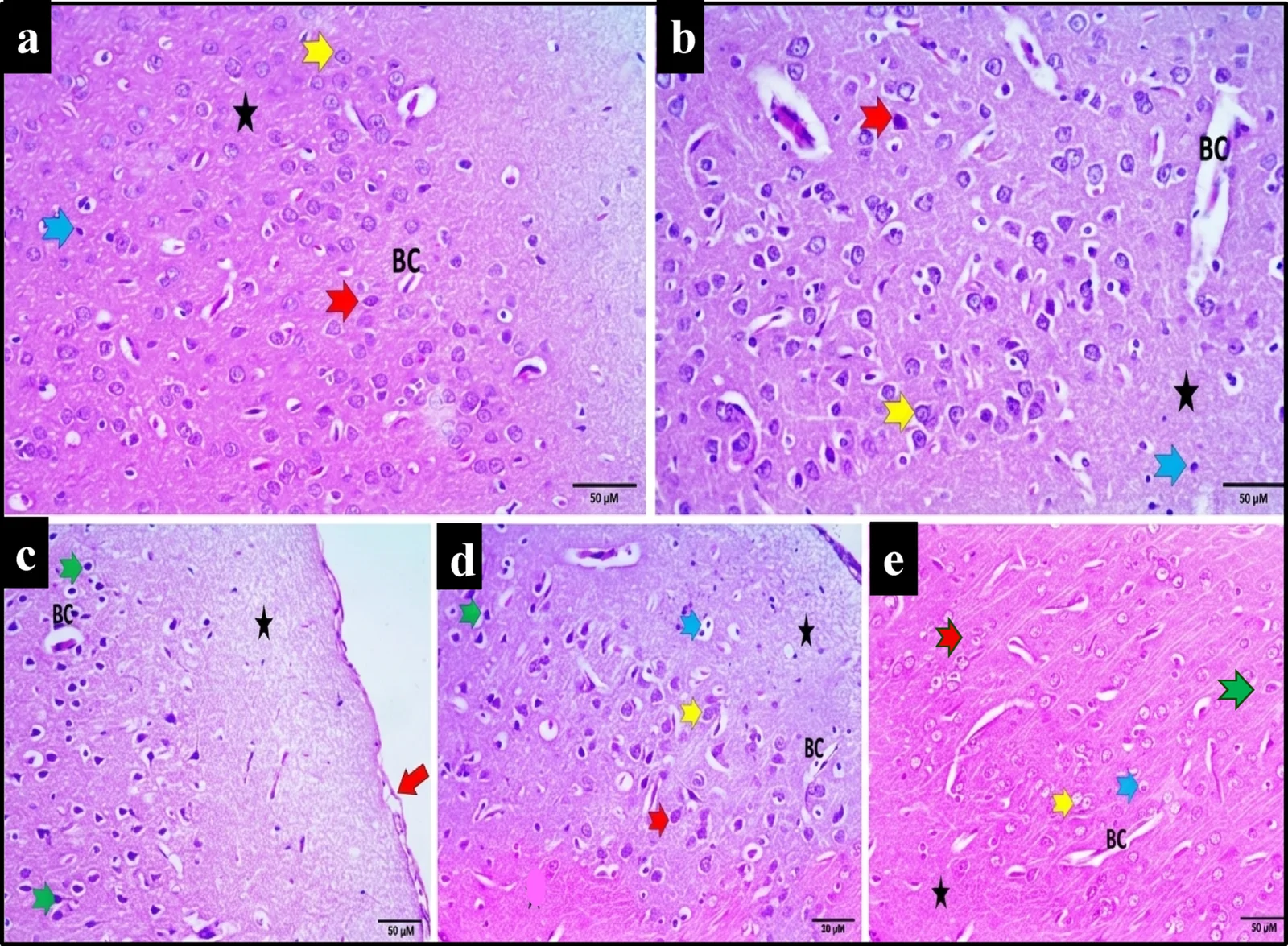
Representative histological changes in cerebral cortex sections of control, QSP, aged, treated, and protective groups. **a** Control group; **b** QSP group; **c** Aged group; **d** Treated group; **e** Protective group. Yellow arrows indicate normal granular cells with rounded pale nuclei; red arrows indicate pyramidal cells; blue arrows indicate neuroglial cells with small dark nuclei; * indicates neuropil; BC indicates blood capillaries; thin arrows indicate irregular capsule; green arrows indicate deeply stained pyknotic nuclei of pyramidal cells. Histopathological lesions were evaluated semi-quantitatively and are presented in Table 5. Original magnification ×400; scale bar = 50 µm

The histological examination of the H&E-stained cerebral cortex section of control and QSP groups showed normal histological architecture of neocortex; normal granular cells with rounded pale nuclei, pyramidal cells, neuroglial cells with small dark nuclei surrounded by normal neuropil. Normal blood capillaries were seen as displayed in Fig. 2a and b, respectively. On the other hand, the cerebral cortex of the aged group exhibited irregular capsule, deeply stained pyknotic nuclei of most granular and pyramidal neurons, dilated blood capillary and mild vacuolated neuropil, as shown in Fig. 2c. The administration of quinoa in the treated and protected groups reduced histopathological alterations in cerebral cortex section of the aged group, as indicated in Table 5. The cerebral cortex section of the treated group manifested moderate improvement of histological architecture of neocortex; most granular cells and pyramidal cells were normal with rounded pale nuclei and, neuroglial cells with small dark nuclei but with perinuclear halo surrounded by mild vacuolated neuropil. Few nuclei of pyramidal cells still have deeply stained nuclei. Normal blood capillaries were seen in Fig. 2d. In the protective group, the cerebral cortex section showed marked improvement of histological architecture of neocortex more or less similar to control group; normal granular cells with rounded pale nuclei, pyramidal cells, neuroglial cells with small dark nuclei but with perinuclear halo surrounded by normal neuropil. Few nuclei of pyramidal cells still have deeply stained nuclei (green bifid arrow). Normal blood capillaries were seen as illustrated in Fig. 2e.

**Table 5:** Semiquantitative histopathological scoring of lesions observed in the cerebral cortex and CA1 hippocampal region

Groups	Neuronal loss Score (0–2)	Neuronal Edema Score (0–3)	Nuclear Pyknosis Score (0–3)	Parenchymal Congestion Score (0–2)
Group (1) Control	0	0	0	0
Group (2) Aged	2	3	3	2
Group (3) QSP	0	0	0	0
Group (4) Treated	1	1	1	1
Group (5) Protected	1	0	0	1

Scores were assigned based on five high-power fields (HPFs)

Histopathological lesions were graded semi-quantitatively as follows:

Neuronal loss: 0 = absent, 1 = mild, 2 = marked

Neuronal edema: 0 = absent, 1 = mild, 2 = moderate, 3 = severe

Nuclear pyknosis: 0 = absent, 1 = mild, 2 = moderate, 3 = severe

Parenchymal congestion: 0 = absent, 1 = mild, 2 = marked

**Fig. 3:**
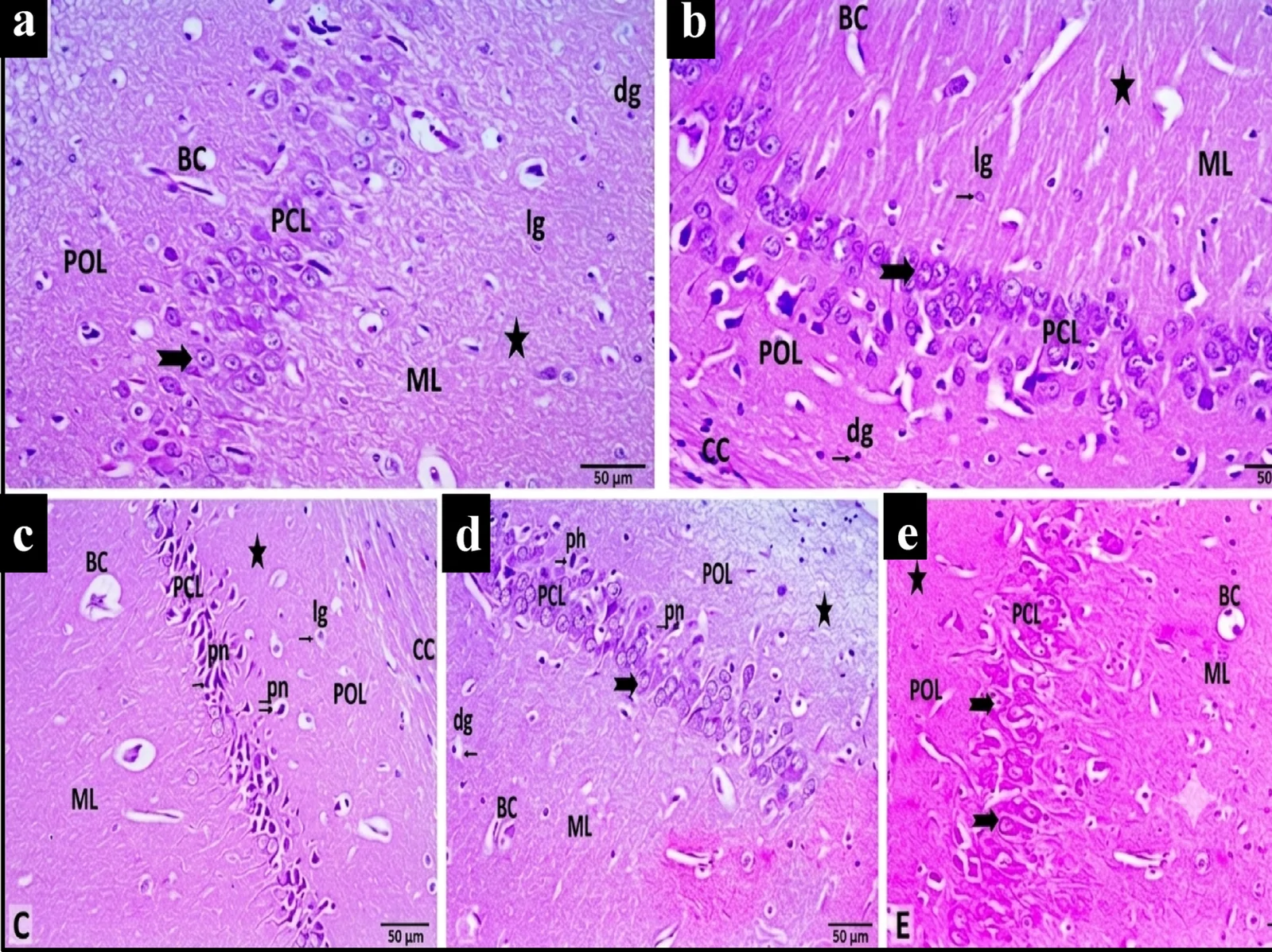
Representative histological changes in the cornu Ammonis 1 (CA1) region of the hippocampus of control, QSP, aged, treated, and protective groups. **a** Control group; **b** QSP group; **c** Aged group; **d** Treated group; **e** Protective group. POL, polymorphic layer; PCL, pyramidal cell layer; ML, molecular layer; BC, blood capillary; * indicates neuropil; bifid arrows indicate pyramidal neurons; dg indicates deeply stained nuclei of glial cells; lg indicates lightly stained nuclei of glial cells; Pn indicates pyknotic nuclei; double arrows indicate scattered pyknotic pyramidal neurons; CC, corpus callosum. Histopathological lesions were evaluated semi-quantitatively and are presented in Table 5. Original magnification ×400; scale bar = 50 µm

A photomicrograph of a section of cornu Ammonis 1 region of hippocampus control and QSP groups manifested well-defined three layers: polymorphic layer (POL), pyramidal cell layer (PCL) and molecular layer (ML). PCL exhibits closely packed cell bodies of the pyramidal neurons that are regularly arranged in 3 to 4 rows and appear small with vesicular nuclei, prominent nucleoli and scanty cytoplasm. The POL and ML display deeply stained and lightly stained nuclei of glial cells with normal blood capillaries. Normal neuropil and corpus callosum (CC) were seen, as shown in Fig. 3a and b, respectively. On the other hand, the aged group showed well-defined three layers; POL, PCL and ML. PCL layer revealed shrunken pyknotic nuclei and Scattered pyknotic pyramidal neurons within POL layer and perinuclear halo around lightly stained nuclei of glial cells and dilated blood capillaries as depicted in Fig. 3c. The administration of quinoa in the treated and protected groups reduced histopathological alterations in the section of cornu Ammonis 1 region of hippocampus of the aged group, as indicated in Table 5. In the treated group, the section indicated moderate improvement formed of well-defined three layers; POL, PCL and ML. PCL exhibit closely packed cell bodies of most pyramidal neurons that are regularly arranged in 3 to 4 rows and appear small with vesicular nuclei, prominent nucleoli and scanty cytoplasm. Few pyramidal cells revealed shrunken pyknotic nuclei and perinuclear halo around deeply stained nuclei of glial cells. Mild vacuolated neuropil and normal blood capillaries, as displayed in Fig. 3d. A photomicrograph of protective group exhibited a marked improvement of histological architecture more or less similar to control group; well-defined three layers; POL, PCL and ML. PCL exhibits closely packed cell bodies of the pyramidal neurons that are regularly arranged in 3 to 4 rows and appear small with vesicular nuclei, prominent nucleoli and scanty cytoplasm. The POL and ML display deeply stained nuclei of glial cells with normal blood capillaries. Normal neuropil was seen as demonstrated in Fig. 3e.

#### Docking studies

**Fig. 4:**
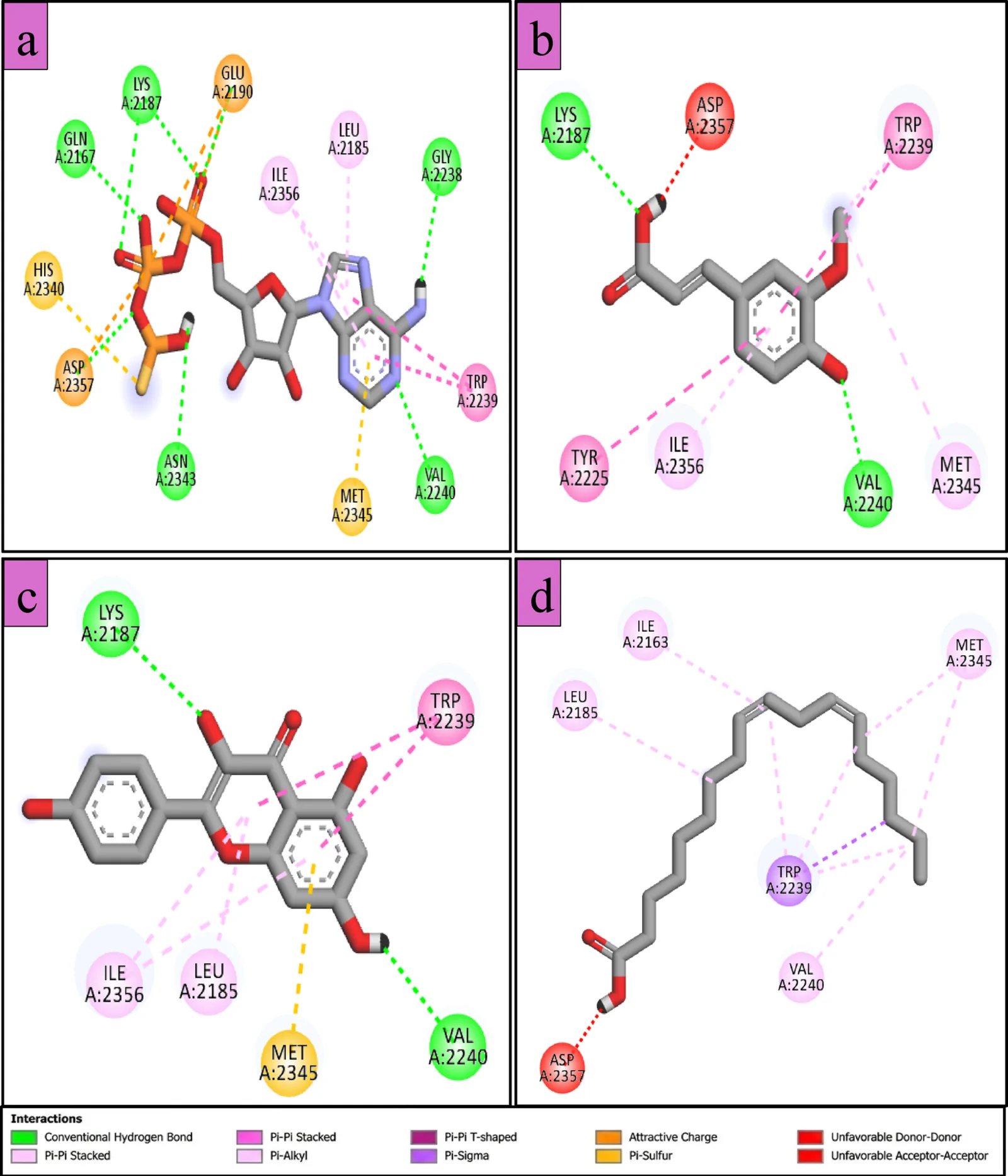
Two-dimensional interaction diagrams of mTOR with **a** The co-crystallized ligand ATP-γS-Mg complex, **b** Ferulic, **c** Kaempferol, and **d** Linoleic acid

**Fig. 5:**
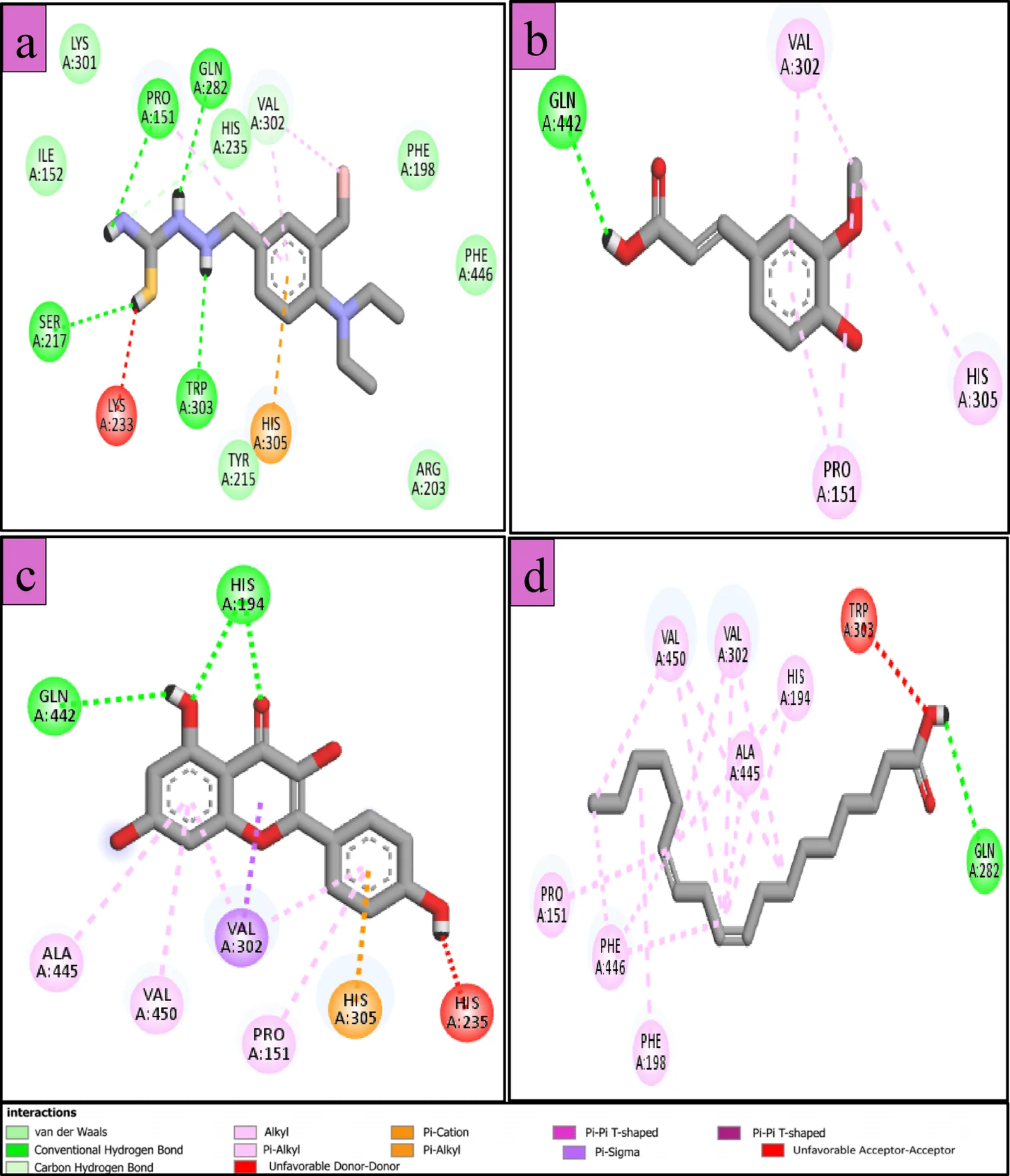
Two-dimensional interaction diagrams of catalase with **a** The co-crystallized ligand LLR, **b** Ferulic, **c** Kaempferol, and **d** Linoleic acid

The docking protocol was initially validated by redocking the corresponding co-crystallized reference ligands into their respective binding sites. The RMSD values between the crystallographic and redocked poses were 2.00 Å for ATP-γS-Mg in the mTOR binding site and 1.80 Å for LLR in the catalase binding site, confirming the reproducibility of the docking protocol. The docking results expressed as binding free energy (ΔG) are presented in Table 6, while the corresponding 2D structures and interacting amino acid residues for all compounds are illustrated in Figs. 4 and 5).

**Table 6:** Binding free energies (*Δ G*) of the investigated ligands

Protein	Ligands	Binding free energy, (*Δ G*) (kcal/mol)
mTOR	Kaempferol	−7.579
	Ferulic acid	−6.581
	Linoleic acid	−5.787
	ATP-γS-Mg complex	−6.575
Catalase	Kaempferol	−7.286
	Ferulic acid	−5.838
	Linoleic acid	−5.600
	LLR	−5.783

#### Molecular dynamics analysis

**Fig. 6:**
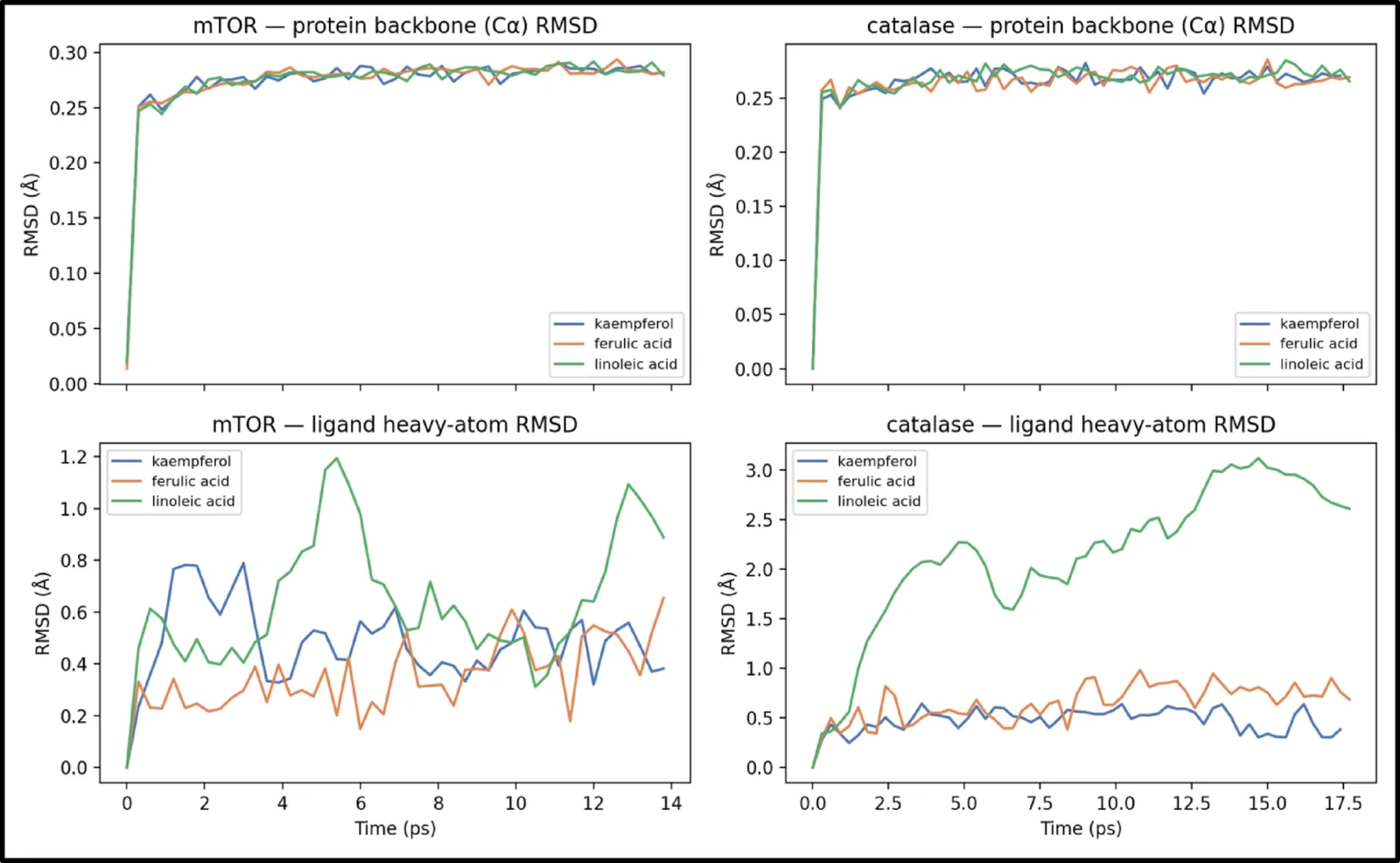
Protein Cα RMSD and ligand heavy-atom RMSD over the molecular dynamics trajectories for the six protein–ligand complexes

**Fig. 7:**
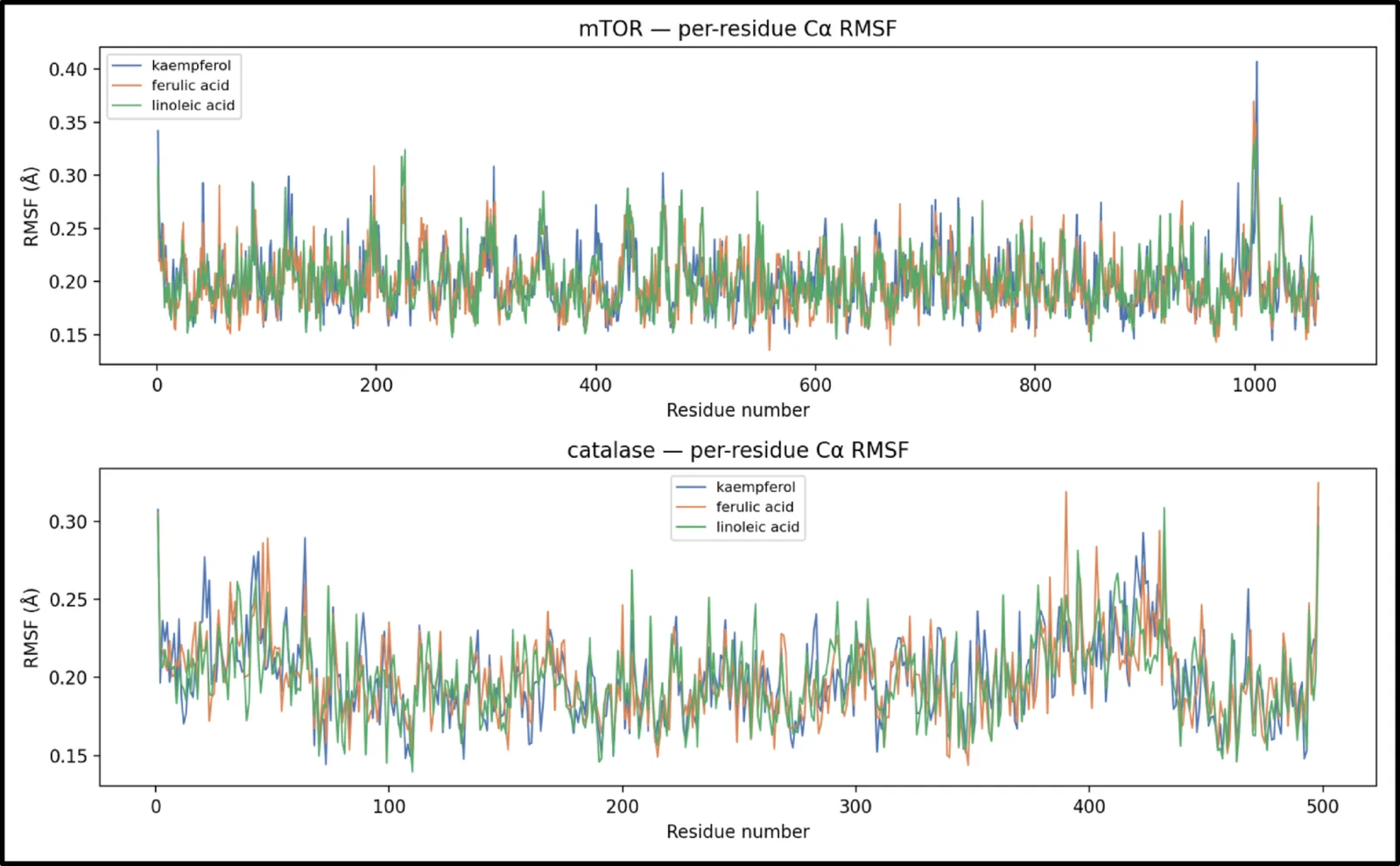
Per-residue Cα RMSF profiles over the molecular dynamics trajectories for the six protein–ligand complexes

**Fig. 8:**
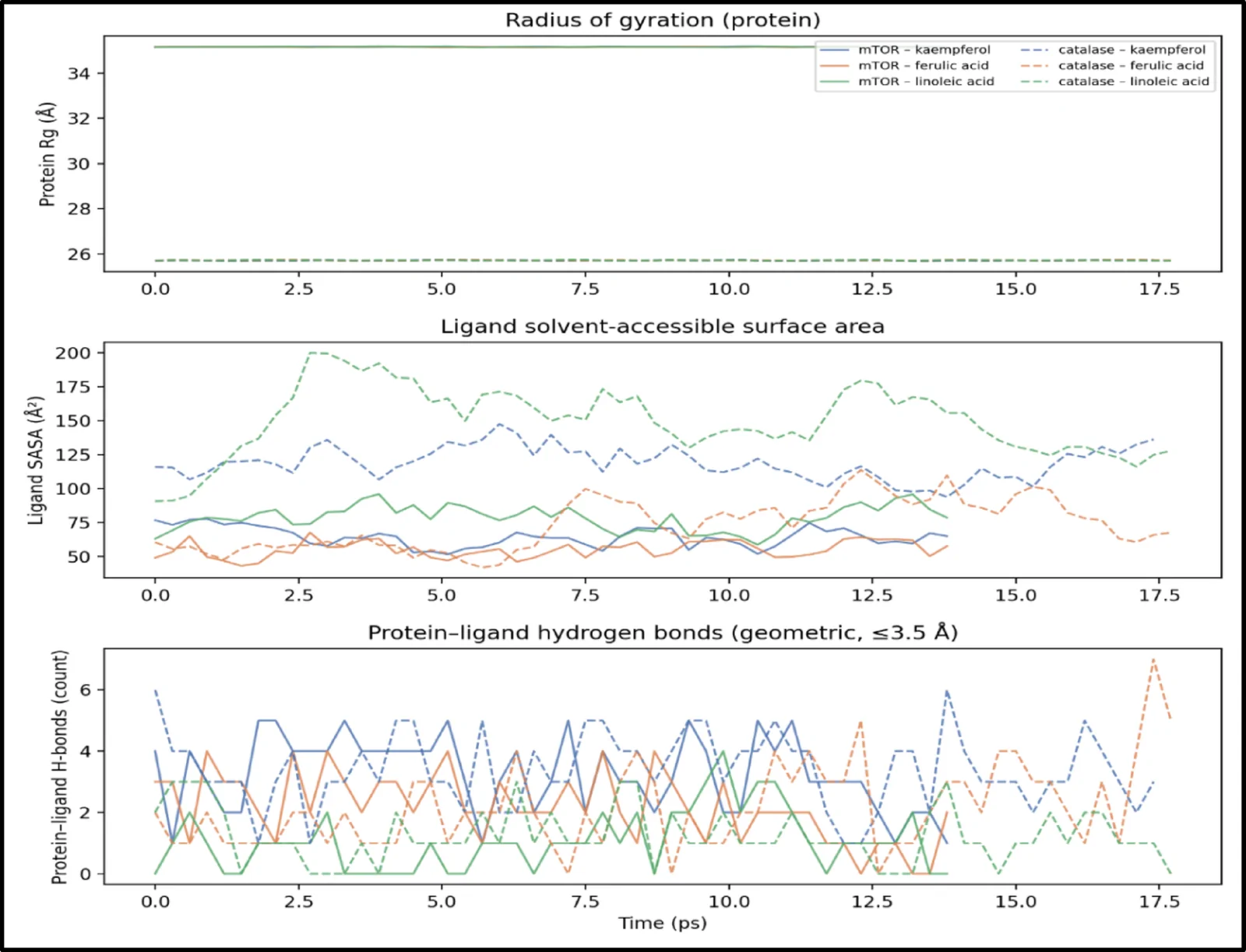
Protein radius of gyration, ligand solvent-accessible surface area (SASA), and protein–ligand hydrogen-bonding interactions over the molecular dynamics trajectories for the six complexes

Short-timescale molecular dynamics analysis showed low protein Cα RMSD values across all complexes, ranging from 0.26 to 0.27 Å, with minimal variation in the radius of gyration, indicating no major structural perturbations over the simulated period. The temporal profiles of protein Cα RMSD and ligand heavy-atom RMSD for the six protein–ligand complexes are shown in Fig. 6. Kaempferol exhibited relatively low ligand RMSD values for both mTOR and catalase (0.48 ± 0.15 and 0.47 ± 0.12 Å, respectively) and showed higher average protein–ligand hydrogen-bonding interactions than the other tested compounds. In contrast, linoleic acid displayed greater ligand mobility, particularly within the catalase complex (2.14 ± 0.73 Å). The per-residue Cα RMSF profiles across the six complexes are presented in Fig. 7, while the trajectory profiles of protein radius of gyration, ligand solvent-accessible surface area (SASA), and protein–ligand hydrogen-bonding interactions are shown in Fig. 8. The trajectory-derived RMSD, RMSF, Rg, SASA, and hydrogen-bonding parameters are summarized in Table 7.

**Table 7:** Trajectory-averaged molecular dynamics parameters for the six protein–ligand complexes

Protein	Ligand	Cα RMSD (Å)	Ligand RMSD (Å)	Rg (Å)	Ligand SASA (Å^2^)	H-bonds
mTOR	Kaempferol	0.27 ± 0.04	0.48 ± 0.15	35.15 ± 0.01	64.40 ± 7.00	3.26 ± 1.21
	Ferulic acid	0.27 ± 0.04	0.35 ± 0.13	35.15 ± 0.01	55.10 ± 6.10	2.17 ± 1.10
	Linoleic acid	0.27 ± 0.04	0.64 ± 0.24	35.15 ± 0.01	78.30 ± 9.00	1.06 ± 0.98
Catalase	Kaempferol	0.26 ± 0.04	0.47 ± 0.12	25.71 ± 0.01	118.70 ± 11.80	3.41 ± 1.19
	Ferulic acid	0.26 ± 0.03	0.65 ± 0.19	25.72 ± 0.01	73.30 ± 18.30	2.20 ± 1.36
	Linoleic acid	0.26 ± 0.04	2.14 ± 0.73	25.72 ± 0.01	149.30 ± 25.40	1.25 ± 0.89

Values are presented as mean ± SD over the analyzed trajectory frames

## Discussion

The brain is a vital organ affected during aging since it controls movement and cognitive activities. In elderly peoples, age-related disorders can cause severe deterioration in memory and cognitive function (Chou et al. 2023).

To evaluate the multi-targeted neuroprotective effect of QSP, the D-gal induced brain senescence model was specifically selected over other chemical-induced neurotoxicity models due to its major methodological and pathophysiological advantages. First, D-gal provides unparalleled fidelity to natural, multi-pathological physiological aging. While acute chemical models like scopolamine primarily induce transient cholinergic dysfunction without mimicking chronic age-related proteinopathy. Also aluminum chloride (AlCl_3_) or sodium azide (NaN_3_) trigger localized chemical-induced neurofibrillary tangles or acute mitochondrial complex IV inhibition respectively (Bayraktaroğlu et al. 2025; Janet Olufunmilayo et al. 2025).Chronic D-gal administration caused metabolic-driven accumulation of advanced glycation end products (AGEs) and sustained ROS that simultaneously activates broad hallmarks of cellular aging, including telomere attrition, autophagic flux impairment (mTOR/LC3B) modulation, and endogenous Tau/ Aβ42 accumulation (Pantiya et al. 2023b). Second, D-gal exhibits superior tolerability and minimal systemic mortality for long-term functional studies. Unlike diabetogenic and severe neurotoxic agents such as streptozotocin which often cause high systemic mortality, severe peripheral organ failure, and metabolic derangements that compromise long-term animal survival, sub-chronic D-gal administration at 250 mg/kg is well tolerated over extended durations (up to 16 weeks) with zero mortality and highly experimental reproducibility(Sedik et al. 2025).

Biochemical alterations in different organs during aging contributed to age-related complications. The brain is particularly vulnerable to oxidative stress because of its high metabolic activity, high content of polyunsaturated fatty acids, and relatively limited antioxidant defenses (Pantiya et al. 2023a).

Quinoa is referred to as a "super food" because of its abundance of beneficial ingredients, which include antioxidant enzymes, fibers, vitamins, minerals, flavonoids, polyphenols, and numerous essential amino acids. Amino acids like lysine and methionine, which are deficient in some cereals, are abundant in its grains (Hussain et al. 2021).

Previous study reported that, quinoa seeds remarkably showed high content of flavonoids (quercetin, kaempferol, and their glycosides), polyphenols (phenolic acids such as ferulic acid, vanillic acid, and their derivatives), vitamin E and carotenoids (Shahzad et al. 2021).

The in vitro antioxidant activity of QEE as inferred from the DPPH radical scavenging activity, indicated that IC_50_ was found to be 5.91 mg/mL. These findings were in harmony with different reports as Park et al. (2017), who demonstrated that quinoa IC_50_ values ranged from 0.25 to 0.47 mg/mL from Korea, USA and Peru. Additionally, Nsimba et al. (2008), who studied quinoa ecotypes from Japan and Bolivia, found IC_50_ values ranging from 0.1 to 7.5 mg/mL for Japanese ecotypes to 0.3–15.8 mg/mL for Bolivia Altiplano. Variations in antioxidant capacity among quinoa ecotypes are expected due to genetic, agrotechnical, and environmental factors affecting its phenolic content. Also, it might be challenging to compare the results of several investigations due to differences in experimental conditions.

Behavior examination by using Y-maze showed that, D-gal and γ-radiation caused a significant reduction in the memory of the animals compared with normal rats. Results from the current study closely correspond with those of Salama and El Shahed (2024), who demonstrated that high doses of D-gal disrupted animals’ ability to learn the Y maze and identify objects. It was found that QSP improved cognitive impairments caused by D-gal and γ-radiation versus untreated group. The data obtained was in accordance with Lan et al. (2025a) who revealed that, quinoa ethanol extract improved cognitive deficits caused by hypobaric hypoxia in rats, which was strongly related to the rich natural polyphenolic compounds. Moreover, Terreros et al. (2024), reported that quinoa consumption mitigated the spatial memory loss by increasing cognitive performance in stressed rats. Also, Souza et al. (2020) indicated that red quinoa seeds alleviated memory impairments caused by scopolamine therapy in rats.

Oxidant/antioxidant imbalance was observed in aging induced rats by D-gal and ɤ-radiation. D-gal metabolites include galactitol and H_2_O_2_ that alter the cellular redox balance. Noureen et al. (2019) found that, superoxide dismutase (SOD), CAT and GSH lower ROS levels which stop DNA, lipids and proteins from oxidation. Furthermore, Chen et al. (2016) found that D-gal administration resulted in a significant oxidative damage in mice's hippocampus as indicated by decreased SOD, glutathione peroxidase (GSH-Px) and CAT activities and an increased MDA level. Additionally, Zhu et al. (2021) proved that exposure to γ-radiation showed a reduction in the levels of GSH, CAT and SOD than usual. The present study was in accordance with the previously mentioned findings in which aging induced rats revealed remarkable reductions in GSH content and CAT activity with elevation in MDA level.

In current study, administration QSP in induced aging rats caused a significant reduction of brain MDA level and increased level of GSH and CAT activity, reflecting its efficacy to prevent D-gal and γ-radiation-induced intracellular oxidative stress. These findings are consistent with Aniess et al. (2020), who reported that the activities of SOD, CAT and GPx increased significantly in rats that fed different doses of red quinoa seeds. Furthermore Li et al. (2018) investigated the ability of quinoa ethanolic extract to inhibit lipid peroxidation and hydroxyl radicals, preserve antioxidant enzyme activity and reduce oxidative stress.

Beyond restoring the antioxidant defense system, attenuation of oxidative stress may exert broader anti-aging effects by protecting DNA telomer from oxidative damage. Telomeric regions are particularly susceptible to ROS-induced lesions, leading to persistent DNA damage signaling and accelerated cellular senescence. According to Xu et al. (2025) excessive oxidative stress preferentially damages telomeric DNA because of its high susceptibility to ROS, thereby accelerating telomere attrition and promoting cellular senescence. Therefore, the reduction in MDA together with the restoration of GSH and catalase activity observed following QSP administration may contribute to preserving telomere integrity and delaying neuronal cellular senescence.

The LC3 protein family is widely used as an autophagy-related marker. During autophagosome formation, cytosolic LC3-I is lipidated to form LC3-II, which is associated with the autophagosome membrane. Therefore, LC3-II levels are commonly assessed as an indicator of autophagy-related activity; however, LC3-II levels alone do not establish autophagic flux (Zhou et al. 2024). In addition, the mTOR acts as a central regulator of autophagy pathway; inhibiting its activity enhances longevity by activating autophagy, which clears out damaged cellular components that contribute to age-related diseases (Mannick and Lamming 2023).

The aging induced rats by D-gal and γ-radiation in the present study showed a significant decrease in LC3B level and a significant increase in mTOR level, indicating that D-gal and γ-radiation suggesting an alteration in autophagy-related signaling. These findings in agreement with (Almeida et al. 2017), who explained that aging causes autophagy to become dysregulated, promoting the accumulation of "garbage" inside the cell. Furthermore, mTOR complexes become overexpressed, causing a rise in aberrant protein aggregates.

In the current study, the rats who received QSP showed a significant restoration in those investigated autophagy-related markers (decrease in mTOR and increase in LC3B levels). Although this data indicated increased autophagosomes formation for sake of cellular recycling, it remains open to the subsequent interpretation of either autophagic modulation or blocked lysosomal degradation. These findings in agreement with those of Koumayha et al. (2026), who observed that quinoa extract administered as a pretreatment and posttreatment for 8 weeks could significantly downregulate the level of mTOR gene expression in dimethylhydrazine (DMH)-induced colorectal cancer models. In addition, Gao et al. (2025), found that quinoa polyphenols increased autophagic flux by increasing LC3B2, ATG4B, and Beclin1. Additionally, Shang et al. (2025) reported that quinoa saponins can activate autophagy in HT-29 cells by regulating the expression of LC3-II and Beclin1. Furthermore, Han et al. (2024) demonstrated that increased Beclin1 and LC3-II expression triggered autophagy.

The Tau protein is a neuronal microtubule-associated protein found in the brain in many isoforms. These are produced by post-translational changes, including phosphorylation, alternative splicing, and intron retention. Its importance is highlighted by its central involvement in Alzheimer's disease (AD). Tau aggregation buildup is a significant pathogenic characteristic of this disease. Given that aging is the key risk factor for Alzheimer's disease, the pathogenic effects of various tau isoforms, particularly phosphorylated ones, may be due to their capability to promote the aging of neurons (Avila et al. 2025). Tau protein detaches from microtubules when it is hyperphosphorylated by GSK-3β, and conformational changes cause Tau protein to form aberrant paired helices and straight filaments, which are then aggregated (Glynn et al. 2024).

Amyloid -beta (Aβ) peptides are proteolytic derivatives of the transmembrane amyloid precursor protein generated by the sequential cleavage by beta- and gamma-secretases enzymes. In healthy brain of all ages, Aβ is present and physiologically essential (containing both Aβ40 and Aβ42) for synaptic plasticity (Cai et al. 2023). Amyloid may increase oxidative stress and trigger tau kinases, resulting in tau alterations that increase pathological aging. Amyloid aggregates produce hyperactivation of neurons, which enhances Tau protein translation, resulting in accelerated cortical aging. Also, deposition of Aβ in the brain can cause oxidative stress, resulting in neurodegeneration and impaired cognitive performance. (Kobayashi et al. 2019; Viña et al. 2024).

In present study, a significant elevation in the levels of tau and Aβ was reported in the aging induced rats by D-gal and γ-radiation, which is in agreement with Rehman et al. (2017), who demonstrated that D-gal caused brain aging by elevating amyloid protein, β-secretase 1, and the advanced glycation end products receptor. Furthermore, Chen et al^30^ revealed that in comparison to normal control, the hippocampus CA1 area of D-gal model mice exhibited considerably higher production of Aβ42 proteins. Also, (Vossel et al. 2015) reported that, brain aging has a larger proportion of Aβ42, which is more likely to form oligomers and fibrils. The most toxic entity is Aβ42 oligomers, which activate glycogen synthase kinase−3β that is involved in tau phosphorylation.

Telomerase, a ribonucleoprotein enzyme complex, is essential for preserving telomere length and genomic stability, which regulates cellular aging and proliferation. Telomerase activity in aging models is essential for studying aging-associated diseases because alterations in telomere length and telomerase activity with age lead to physiological deterioration and age-related disorders (Rossiello et al. 2022). Reduction in telomerase activity was observed in rats induced aging by D-gal and ɤ-radiation compared with control. This is agreed with Zhu et al. (2014), who claimed that administration of D-gal significantly reduced the telomere lengths and the telomerase activity when compared to the control rats.

Importantly, the observed restoration of brain telomerase activity in QSP-treated rats is strongly supported by the concurrent attenuation of oxidative stress and positive modulation of autophagic flux. Oxidative damage induced by D-gal and ɤ-radiation is a primary driver of telomere shortening and telomerase inactivation. By elevating catalase activity and GSH levels, QSP protects telomeric DNA integrity. Furthermore, the robust down-regulation of mTOR and upregulation of LC3B reflect a restored autophagic mechanism that clears cellular debris and damaged organelles, maintaining the cellular energy homeostasis required to sustain telomerase function and prevent neuronal senescence (Wei et al. 2025; Rejili et al. 2026).

Stability of telomerase activity may also interrupt the progression of stress-induced cellular senescence by preventing critical telomere dysfunction and limiting activation of the p53/p21 signaling cascade. Xu et al. (2025) highlighted that preservation of telomere integrity limits persistent DNA damage responses and subsequent activation of senescence-associated pathways, including the p53/p21 axis. Although these downstream mediators were not directly investigated in the current study, preservation of telomerase activity together with attenuation of oxidative stress suggests that QSP may indirectly protect neuronal cells against senescence-associated molecular events.

In this study, it was found that, QSP administration effectively ameliorated abnormalities in the levels of tau and Aβ and telomerase activity of treated rats towards the normal levels in comparison with aged rats. The protective effect of QSP may be related with its high content of flavonoid and polyphenol, given that several studies have reported the involvement of flavonoids may prevent amyloid plaque development by binding to Aβ and preventing aggregation (Hole and Williams 2020). Additionally, polyphenols exhibit neuroprotective qualities by preventing tau hyperphosphorylation and amyloid-beta (Aβ) aggregation (Freyssin et al. 2018; Han et al. 2023). Furthermore, polyphenols may contribute to telomere preservation and attenuate telomere shortening, potentially through their antioxidant and anti-inflammatory effects (D’Angelo 2023).

Despite extensive research on neurodegenerative disorders, significant gaps remain regarding multi-targeted therapeutic options that can simultaneously counteract proteinopathy, telomere dysfunction, and impaired autophagic flux under combined environmental and metabolic stressors. The current study bridges these gaps by demonstrating that QSP not only quenches oxidative stress but actively resets autophagic mechanisms (via mTOR/LC3B modulation) and reduces neurotoxic Tau and Aβ42 accumulation. These findings provide a holistic biochemical rationale for integrating functional dietary interventions into neuroprotective strategies against complex brain senescence.

The simultaneous modulation of mTOR and LC3B together with the reduction in Aβ42 and Tau suggests a functional crosstalk between autophagy and protein homeostasis during brain aging. Hruby and Higuchi-Sanabria (2025) emphasized that impaired autophagy promotes the accumulation of dysfunctional mitochondria and neurotoxic protein aggregates, thereby accelerating neuronal senescence, whereas restoration of autophagic activity enhances mitochondrial function. Therefore, the coordinated regulation of autophagy-related markers and neurodegenerative proteins observed following QSP administration provides a plausible mechanistic explanation for its neuroprotective effects.

The biochemical findings were supported by histological examination that revealed that administration of QSP in the treated and protected groups reduced histopathological alterations in cerebral cortex and hippocampus of aged group.

To further support the biochemical findings and histopathological examination, molecular docking analysis was performed against molecular targets directly related to the investigated pathways. Since Tau protein and amyloid-β are aggregation products rather than conventional druggable enzymatic targets, docking was conducted against mTOR, a master negative regulator of autophagy, and catalase, a key antioxidant enzyme involved in cellular defense against oxidative stress.

The docking analysis predicted favorable binding affinities of the major quinoa constituents, namely kaempferol, ferulic acid, and linoleic acid, toward the selected targets, suggesting potential interactions with proteins involved in antioxidant defense and autophagy-related pathways. Among the tested compounds, kaempferol showed the strongest and most consistent binding profile across both targets. This behavior may be attributed to its flavonoid structure, which combines a planar aromatic scaffold capable of hydrophobic and π-interactions with multiple hydroxyl groups that facilitate hydrogen-bond formation within the binding pockets (Calderon-Montano et al. 2011).

In the mTOR active site, kaempferol displayed hydrogen-bond interactions with Lys2187 and Val2240, in addition to π–π stacking with Trp2239 and several hydrophobic contacts, suggesting a potentially favorable binding mode within the predicted binding pocket. Against catalase, kaempferol exhibited the highest binding affinity among the tested compounds, with a docking score of −7.286 kcal/mol, surpassing that of the co-crystallized ligand LLR (− 5.783 kcal/mol). The interaction profile revealed conventional hydrogen bonds with Gln242 and His194, together with π-interactions and hydrophobic contacts involving Val302, Pro151, Ala45, Val450, and His305, suggesting favorable binding within the enzyme pocket. In comparison, ferulic acid and linoleic acid exhibited lower binding affinities, with docking scores of −5.838 and −5.600 kcal/mol, respectively.

In contrast, ferulic acid exhibited fewer stabilizing interactions, whereas linoleic acid interacted predominantly through hydrophobic contacts with limited hydrogen-bond formation and even showed an unfavorable interaction within the mTOR binding pocket. Therefore, the superior docking performance of kaempferol appears to be related to its ability to simultaneously establish hydrogen-bonding, aromatic stacking, and hydrophobic interactions within the target proteins.

The molecular dynamics analysis provided an additional structural perspective on the docking predictions. The low protein Cα RMSD and stable radius of gyration over the simulated period indicated the absence of major structural perturbations, while the relatively low ligand RMSD and higher hydrogen-bonding frequency observed for kaempferol were consistent with its favorable predicted docking profile. Nevertheless, these findings should be interpreted as preliminary short-timescale computational evidence, as the simulations were performed over a limited timescale and under simplified solvent conditions. Therefore, they support the plausibility and short-timescale structural persistence of the predicted interaction modes but do not establish biological binding or pharmacological activity.

## Limitations and future perspectives

While the current study comprehensively highlights the multi-targeted efficacy of QSP against brain senescence via appreciated alterations in the autophagic flux signaling markers (mTOR/LC3B), telomerase restoration, and reduction of Tau and Aβ42 accumulation, certain limitations and prospective avenues warrant future investigation. This work focused primarily on cellular senescence hallmarks rather than complete neurotransmitter profiling; thus, future studies evaluating the impact of QSP on the neuro-inflammatory markers, cholinergic system—including acetylcholinesterase (AChE) dynamics—and other neurotransmitter pathways will further complement these mechanistic findings. Furthermore, synaptic integrity in correlation to mitochondrial dysfunction is to be investigated as mitochondria are heavily localized at nerve endings to power neurotransmission. Clinical translation through human intervention trials is essential to establish the pharmacokinetics, bioavailability, and therapeutic efficacy of standardized QSP in age-associated neurodegenerative disorders. This rodent-to-human translation requires careful metabolic scaling as raw rodent data cannot be directly extrapolated to human clinical efficacy. Integrating advanced cell-type-specific transcriptomics (scRNA-seq) could further delineate the precise cellular targets of QSP within the central nervous system. While the current findings demonstrate the modulation of core autophagic markers, further in-depth investigations are required to comprehensively elucidate the complete autophagic pathway. In the present study, cognitive evaluation was focused on short-term spatial working memory using the Y-maze spontaneous alternation test. Even though the Y-maze offers a non-invasive, low-stress, and validated measure of prefrontal-hippocampal function, future studies incorporate additional behavioral assays will further elucidate the broader neurobehavioral profile of quinoa extract. In silico simulations provide structural insights into potential ligand–protein interactions, but they do not account for critical physiological variables such as blood–brain barrier permeability, systemic metabolism, or functional cell-based kinetics. Therefore, these computational findings serve as a foundation for future target-validation and bioactivity assays.

## Conclusion

In conclusion, this study provides the first comprehensive evidence that phytochemical-based QSP effectively attenuates D-gal and γ-radiation-induced brain cellular senescence and aging. Beyond its conventional antioxidant capacity, QSP exerts a multi-targeted neuroprotective strategy by significantly restoring telomerase activity, reducing neurotoxic Tau protein and Aβ42 accumulations, and modulating autophagic flux via mTOR and LC3B pathways. Furthermore, molecular docking highlights kaempferol as a potential lead bioactive compound driving these regulatory effects against mTOR and catalase. Collectively, these novel mechanistic insights underscore the therapeutic promise of QSP as promising functional nutritional intervention to counteract age-associated neurodegenerative disorders.

## Supplementary Information

Below is the link to the electronic supplementary material.

Supplementary Material 1

## Data Availability

All Data were provided within the manuscript.
