# Supplementary material for: Evaluation the role of quinoa seeds in attenuation the brain cellular senescence and aging induced by D-galactose and γ-radiation in rats: insights into autophagy, telomerase activity, amyloid-β and tau proteins
Source: Inflammopharmacology. 2026 Sep 19;34(10):6819–38. doi: 10.1007/s10787-026-02377-1 (PMC13675366; doi:10.1007/s10787-026-02377-1)
Supplement: Supplementary file 1 [file 10787_2026_2377_MOESM1_ESM.docx]

**Supplementary Figure S1**. Research Ethics Committee approval certificate for animal study.

**Hippocampus**

**Supplementary Figure S2.** Representative histological sections of the hippocampus in the control group.

**Supplementary Figure S3.** Representative histological sections of the hippocampus in the QSP-treated group.

**Supplementary Figure S4.** Representative histological sections of the hippocampus in the aging group.

**Supplementary Figure S5.** Representative histological sections of the hippocampus in the treatment group.

**Supplementary Figure S6.** Representative histological sections of the hippocampus in the protective group.

**Cerebral cortex**

**Supplementary Figure S7.** Representative histological sections of the cerebral cortex in the control group.

**Supplementary Figure S8.** Representative histological sections of the cerebral cortex in the QSP-treated group.

**Supplementary Figure S9.** Representative histological sections of the cerebral cortex in the aging group.

**Supplementary Figure S10.** Representative histological sections of the cerebral cortex in the treatment group.

**Supplementary Figure S11.** Representative histological sections of the cerebral cortex in the protective
